# Effects of α-amylase supplementation on production performance, blood metabolites, nutrient digestibility, and rumen fermentation parameters of Holstein dairy cows in late lactation

**DOI:** 10.3389/fvets.2025.1629571

**Published:** 2025-07-22

**Authors:** Mengen Zhang, Guodong Li, Dian Wang, Shiqin Wang, Hongsheng Du, Rubing Lan, Yiming Xu, Hongkai Liu, Yingli Li

**Affiliations:** ^1^Youran Dairy Co., Ltd., Hohhot, China; ^2^College of Animal Science, Anhui Science and Technology University, Chuzhou, China

**Keywords:** α-amylase, dairy cows in late lactation, production performance, blood metabolites, rumen fermentation

## Abstract

Current research on dairy cows primarily focuses on peak lactation, with limited exploration of late lactation. This study investigated the effects of α-amylase supplementation on production performance, blood metabolites, nutrient digestibility, and rumen fermentation in late-lactation Holstein cows. Thirty cows (average milk yield: 37.48 ± 1.63 kg; parity: 2.44 ± 0.70; lactation days: 210.17 ± 2.20) were randomly divided into two groups: the control group (CON) received a basal diet, while the experimental group (AM) was supplemented with 15 g/day α-amylase for 7 weeks (1-week adaptation + 6-week trial). Results showed that α-amylase significantly increased milk yield, energy-corrected milk (ECM), and milk protein yield (*p* < 0.01) and improved fat-corrected milk (*p* < 0.05). Milk protein content, total solids, and milk fat yield also tended to rise (*p* = 0.061, *p* = 0.067, *p* = 0.091, respectively). No significant differences were observed in dry matter intake (DMI), feed efficiency, or somatic cell count. Serum amylase concentration increased markedly in the AM group (*p* < 0.01), while other blood parameters remained unchanged. Starch digestibility improved significantly (*p* < 0.05), and neutral detergent fiber (NDF) digestibility showed a positive trend (*p* = 0.063). Rumen propionate concentration rose significantly (*p* < 0.05), with no major changes in pH, ammonia nitrogen, or acetate-to-propionate ratio. In conclusion, α-amylase supplementation in late lactation enhances nutrient digestibility, modulates rumen fermentation, and improves production performance, offering metabolic regulation potential for extending high productivity in late-stage lactation.

## 1 Introduction

Facing climate change, volatile agricultural prices, and a complex economy, the dairy industry faces unprecedented challenges: rising costs and weak demand. As breeding costs grow and consumer demands diversify, improving efficiency is key for boosting productivity and cutting costs with limited resources. Starchy grains (corn, wheat, barley)–60–70% of diets—are primary energy sources. Increasing dietary starch is common to meet high-yield needs, but cows' limited endogenous amylase leads to incomplete starch digestion, especially with high intake. As a result, some un-digested starch enters the hindgut, causing microecological disorders and hindgut acidosis (1). Therefore, supplementation of exogenous amylase to enhance starch decom-position and improve the nutrient absorption capacity of dairy cows is a potential strategy to improve dairy cattle production efficiency. α-Amylase, an enzyme specifically hydrolyzing α-1,4-glycosidic bonds, catalyzes starch into maltose, oligosaccharides and dextrins to enhance starch digestibility and reduce fecal starch loss (2). Since the production efficiency and technological development speed in the poultry and monogastric animal industries are usually faster than those in the dairy cattle industry, relevant technologies are adopted earlier to improve efficiency. Therefore, α-amylase is commonly used in poultry (3) and monogastric animal (4) production. With in-depth research, this technology has gradually been applied to dairy cattle production to further improve its production efficiency. Studies on dairy cows in the early lactation period have found that when the dose of amylase is 1.0 g/kg DM, the milk yield and starch digestibility increase significantly, and there is no significant effect on dry matter, fiber digestibility, and milk components (5, 6). In dairy cows in the mid-lactation period, it has been found that supplementation of amylase to the diet has a trend of increasing the digestibility of dry matter and crude protein, can increase the concentrations of acetate, butyrate, and branched-chain fatty acids in the rumen, while reducing the concentration of NH3–N. In addition, it also improves the effect of nitrogen transfer to milk (7). In studies on different starch concentrations and digestive enzymes, it has also been found that supplementation of amylase can optimize the diet structure, improve rumen fermentation, reduce urinary nitrogen excretion, and thus enhance the overall production efficiency of dairy cows (2, 8).

Currently, most studies focus on the production performance of dairy cows in the peak lactation period, while relatively few studies focus on dairy cows in the late lactation period. For dairy cows in the late lactation period, their physiological conditions are special. The mammary gland tissue of dairy cows in the late lactation period is in a state of fatigue, resulting in a decline in production performance (9). Compared with dairy cows in the peak lactation period, the milk quality of dairy cows in the late lactation period improves, and the body condition continues to recover, but milk production decreases. Dairy cows in the late lactation period not only need to meet the nutritional needs of lactation but also consider the needs of pregnancy. When the nutrition of dairy cows is unbalanced, they may suffer from metabolic diseases (such as fatty liver, ketosis, retained placenta, etc.) during calving (10). Therefore, how to improve the digestibility and utilization rate of nutrients through nutritional regulation while ensuring the health of dairy cows has become a key issue in the feeding and management of dairy cows in the late lactation period. Under this background, this experiment aims to investigate the effects of dietary α-amylase supplementation on production performance, blood biochemical indices, nutrient digestibility, and rumen fermentation parameters in dairy cows during late lactation. Through this research, we aim to optimize feeding management strategies for dairy cows in the late lactation phase, thereby improving their health status and productive performance.

## 2 Materials and methods

### 2.1 Experimental design and treatments

This experiment was approved by the Laboratory Animal Management and Ani-mal Ethics Committee of Anhui Science and Technology University (No.2025073). All experimental procedures were carried out in strict accordance with the guidelines established by the Animal Ethics Research Committee.

The α-amylase product is primarily composed of xylanase (derived from fermentation and extraction of Aspergillus oryzae), with α-amylase (on a dry basis) showing an activity of 636 U/g. Its carrier is brewer's yeast powder, and the product is provided by Beijing Alltech Biological Products Co., Ltd.

Thirty dairy cows in late lactation with a milk yield of (37.48 ± 1.63) kg, parity of (2.44 ± 0.70) lactations, lactation days of (210.17 ± 2.20) days, and good health were selected for the experiment. A completely randomized design was used, and the cows were divided into 2 groups of 15 cows each. The control group (CON group) was fed a basal diet, and the experimental group (AM group) was fed the basal diet supplemented with 15 g/day of α-amylase. The supplementation dosage was determined based on the manufacturer's recommendations and previous research findings (11). The experiment lasted for 7 weeks, including a 1-week adaptation period and a 6-week formal trial period. The feeding standards and methods were based on the ranch management standards. The cows had free access to feed and water. They were fed 3 times a day (at 06:00, 12:00 and 18:00) and milked 4 times a day (at 06:30, 12:30, 18:30, and 22:30). The cows were raised in a dry, ventilated, and comfortable environment. The composition and nutrient levels of the basal diet are shown in Table 1.

**Table 1:** Composition and nutrient levels of the basal diet (dry matter basis %).

**Ingredients**	**Content**	**Nutrient levels^2^**	**Content**
Whole corn silage	50.93	NEL/ (Mcal/kg)	1.72
Alfalfa hay	6.27	DM	49.18
Permix^1^	5.09	CP	17.49
Steam flaked corn	6.66	EE	5.58
Corn starch	5.68	Strach	28.02
High moisture content corn	1.96	ADF	16.34
Soybean meal	5.29	NDF	25.68
Extruded soybean	0.59		
Corn gluten meal	0.59		
Double-low rapeseed meal	1.76		
Cottonseed	2.94		
Beer grains	9.79		
Molasses	1.96		
NaHCO_3_	0.20		
Fat powder	0.20		
Urea	0.10		

^1^The premix provided the following per kg of the diet: VA 100000 IU, VD3 280000 IU, VE 2000 IU, Cu 280 mg, Mn 750 mg, Zn 1000 mg, Co 14 mg.

^2^NEL was a calculated value, while the other nutrient levels were measured values.

### 2.2 Sample collection and processing

#### 2.2.1 Collection of diet samples and fecal samples

On the last day of each week during the experimental period, dairy cow diet samples were collected using the quartering method and subsequently placed in an oven at 65°C for 48 h until a constant mass was achieved. The digestion experiment was carried out by the acid-insoluble ash method. During the last 5 days of the trial period, fecal samples were collected rectally for 5 consecutive days, and the fecal samples collected over these 5 days were divided into two portions. One part was fixed for nitro-gen by adding 20 mL of 10% H_2_SO4 to every 100 g of fresh feces to determine the protein content in feces. The other part was put into a self-sealing bag without treatment to determine the contents of other nutrients in feces. Each part was not < 300 g and stored at −20°C for later analysis.

#### 2.2.2 Collection of milk samples

During the experimental period, the milk yield of each cow was recorded daily through the GEA circular turntable milking system, and the average daily milk yield of each group was calculated accordingly. The milk samples were collected on the last day of each week during the trial period. Samples were collected 3 times a day (at 07:00, 14:00, and 20:00), and divided into 50 mL collection tubes in a 4:3:3 ratio and added with potassium dichromate preservative according to the ratio, then stored at +4 °C for later analysis.

#### 2.2.3 Collection of rumen fluid samples

At the end of this experiment, 9 cows were randomly selected from each group. Before the morning feeding, rumen fluid was collected using a flexible oral gastric tube with a metal filter. About 50 mL of the newly collected sample was discarded to avoid saliva contamination. Then, about 300 mL of rumen fluid was extracted, filtered through 4 layers of gauze, and divided into centrifuge tubes. Part of it was used for pH determination, and the rest was divided into 15 mL sterile centrifuge tubes and stored in liquid nitrogen for the determination and analysis of rumen fermentation parameters. The oral gastric tube was cleaned before taking samples from the next animal.

### 2.3 Determination indicators and methods

#### 2.3.1 Production performance

During the experiment, the milk yield and average dry matter intake were measured and recorded daily. Diet samples were collected, and dried in an oven at 105°C for 4 h until constant weight measured the dry matter content. The feeding amount and remaining amount of feed were collected, and the dry matter intake was calculated.


$$\begin{array}{l} \text{Feed efficiency}=\text{Average daily feed intake}/\\ \text{ Average daily milk yield}. \end{array}$$

#### 2.3.2 Determination of milk components

The milk samples were collected on the last day of each week during the trial period. The samples were mixed in a 4:3:3 ratio according to the morning, noon, and evening samples, added with potassium dichromate preservative, and stored in 50 mL centrifuge tubes in a refrigerator (+4°C) for the determination of milk components (milk fat, milk protein, total solids, and somatic cell count). The components and con-tents of milk were determined using a FOSS Milk Oscan™ FT+ automatic milk analyzer (FOSS, Hillerød, Denmark). The calculation formulas for fat-corrected milk (FCM) and energy-corrected milk (ECM) refer to the literature (12) and are as follows:


$$\begin{array}{l} \text{FCM}=\;\left[0.\text{4323}\times \text{Milk yield }\left(\text{kg}/\text{d}\right)\right]\\ \;+\left[\text{16}.\text{216}\times \text{Milk fat yield }\left(\text{kg}/\text{d}\right)\right].\\ \text{ECM}=\;\left[0.\text{327}\times \text{Milk yield }\left(\text{kg}/\text{d}\right)\right]\\ \;+\;\left[\text{12}.\text{95}\times \text{Milk fat yield }\left(\text{kg}/\text{d}\right)\right]\\ \;+\left[\text{7}.\text{2}0\times \text{Milk protein yield }\left(\text{kg}/\text{d}\right)\right]. \end{array}$$

#### 2.3.3 Determination of blood indicators

The blood samples were collected from the caudal vein of the cows before morning feeding on the last day of each week during the trial period. Blood was drawn into 10-mL heparinized tubes, centrifuged at 3,500 rpm for 10 min, and serum was subsequently harvested. Aliquots (2 mL) of serum were transferred to cryogenic tubes and stored at −20°C for subsequent analysis. The detection of blood glucose (GLU), insulin (INS), glucagon (GCG), and amylase was performed using commercial kits (provided by Ningxia Haobiao Testing Research Institute Co., Ltd.), while the detection of urea nitrogen was carried out using an automatic biochemical analyzer (TBA-120FR Auto Clinical Chemistry Analyzer, Toshiba Corporation, Tokyo, Japan).

#### 2.3.4 Determination of nutrient digestibility

The apparent digestibility of nutrients was determined by the endogenous indicator acid-insoluble ash method with reference to the literature (13). The determination of starch content was carried out according to (11) the method in the literature.

The calculation formula for the apparent digestibility of nutrients is as follows:


$$\begin{array}{l} \text{Apparent digestibility}\\ \text{ of nutrients }\left(\%\right)=\text{1}00-[\text{1}00\times (\text{Acid}\\ \;-\text{insoluble ash content in diet }/\text{ Acid}\\ \;-\text{insoluble ash content in feces})\\ \;\times (\text{Nutrient content in feces}/\\ \text{ Nutrient content in diet})]. \end{array}$$

#### 2.3.5 Determination of rumen fermentation parameters

On the last day of the experiment, 9 cows were randomly selected from each group and rumen fluid was collected before morning feeding to determine pH. The pH of the rumen was measured using a pH meter (PHS-10 portable pH meter, Chengdu Century Ark Technology Co., Ltd.). The pH meter was calibrated with appropriate calibration solutions before measurement. The methods of He et al. (14) were referred to for the determination of NH3–N and VFA. Specifically, NH3–N was deter-mined by the phenol-hypochlorous acid colorimetric method. Volatile fatty acids (VFA) were determined by gas chromatography-mass spectrometry.

#### 2.3.6 Economic benefit analysis

Based on the average daily dry matter intake of dairy cows, milk yield, and the selling price of fresh milk, feed costs and milk production revenue were calculated systematically, ultimately deriving the overall economic benefit. The calculation formulas followed the methodologies documented in Reference (15).


$$\begin{array}{l} \text{Milk production revenue}\\ \;\left[\text{CNY}/\;\left(\text{cow}\cdot \text{day}\right)\right]\;=\text{Average daily milk yield}\\ \;\times \text{Fresh milk price per kilogram}.\\ \text{ Feed cost}\\ \;[\text{CNY}/\;\left(\text{cow}\cdot \text{day}\right)]=\text{Average daily dry matter intake}\\ \;\times \text{TMR dry matter price per kilogram}.\\ \text{ Net Economic Benefit}\\ \;\left[\text{CNY}/\;\left(\text{cow}\cdot \text{day}\right)\right]\;=\text{Milk Output Revenue}\\ \;-\text{Feed Input Cost}. \end{array}$$

## 3 Statistical analysis

Before analysis, the normality of data was first tested using the Shapiro-Wilk test, and the homogeneity of variance was examined via the Levene test with SPSS (Windows version 23.0; SPSS Inc., Chicago, USA). If data met the assumptions of normality and homogeneous variance, independent samples *t*-test was used for significance analysis. Use the PROC MIXED model in SAS 9.4 software (SAS Inst. Inc., Cary, NC, USA) to analyze dry matter intake (DMI), milk yield, FCM, ECM, FCM/DMI, ECM/DMI, feed efficiency, SCC, milk composition, and blood indicators in dairy cows. The model includes fixed effects for treatment, time, and treatment × time interaction, with random effects for individual animals. The data was expressed as means and standard errors of the means (SEMs). The significance level of *p* < 0.05 was considered significant, *p* < 0.01 was considered extremely significant, and 0.05 ≤ *p* < 0.10 indicated a significant trend.

The PROC MIXED model is:


$$\begin{array}{l} {\text{Y}}_{ijk}=\mu +\beta \text{1}\cdot \text{Tr}{\text{t}}_{i}+\beta \text{2}\cdot \text{Tim}{\text{e}}_{j}+\beta \text{3}\cdot \left(\text{Tr}{\text{t}}_{i}\times \text{Tim}{\text{e}}_{j}\right)+u_{k}\\ \;+\varepsilon_{ijk.} \end{array}$$

μ: Overall mean, βi: Treatment effect (Trt), β2: Time effect (Time), β3: Interaction effect (Trt × Time), *u*_*k*_: Random intercept for the k-th animal, ε_*ijk*_:Residual error term, Y_*ijk*_: Observation for the *k*-th animal at the *j*-th time point under the *i*-th treatment, Trt_*i*_: Treatment indicator variable, Time_*j*_: Time point indicator variable.

## 4 Results

### 4.1 Effects of dietary α-amylase supplementation on production performance of dairy cows

As shown in Table 2, compared to CON, α-amylase supplementation in dairy cows significantly increased milk yield and ECM, (*p* < 0.01), improved milk protein yield and FCM (*p* < 0.05), and showed trends toward higher milk protein, total solids, and milk fat yields (*p* = 0.061, *p* = 0.067, *p* = 0.091). No significant differences were observed in DMI, FCM/DMI, ECM/DMI, feed efficiency, milk fat%, or SCC (*p* > 0.05). Regarding temporal effects, experimental week significantly affected feed intake, milk yield, FCM, ECM, FCM/DMI, ECM/DMI, milk fat%, total solids, and milk fat yield (*p* < 0.01), while milk protein% (*p* < 0.05) and feed efficiency (*p* = 0.090) showed marginal trends. Milk protein yield and SCC remained stable over time (*p* > 0.05). The interaction between the experimental treatment and experimental weeks had no significant effect on dairy cows (*p* > 0.05).

**Table 2:** Effects of dietary α-amylase supplementation on feed intake, milk yield, and milk composition of dairy cows.

**Items**	**Treatments** ^**1**^		**SEM**	***p*** **-value**		
	**CON**	**AM**		**Trt** ^2^	**Time**	**Trt** × **Time**
Feed intake (kg/d)	27.08	27.48	0.19	0.155	0.001	0.990
Milk yield (kg/d)	37.6^c^	38.24^a^	0.14	0.005	0.001	0.915
FCM (kg/d)	38.80^b^	39.68^a^	0.27	0.047	0.002	0.696
ECM (kg/d)	38.91^c^	39.82^a^	0.19	0.007	0.007	0.728
FCM/DMI (kg/d)	1.43	1.43	0.01	0.907	0.003	0.872
ECM/DMI (kg/d)	1.44	1.44	0.01	0.942	0.002	0.870
Feed efficiency	1.39	1.39	0.01	0.825	0.090	0.985
Milk fat (%)	3.64	3.71	0.04	0.312	0.001	0.604
Milk protein (%)	3.29	3.34	0.02	0.061	0.013	0.738
Total solids (%)	11.99	12.18	0.06	0.067	0.002	0.734
Milk fat yield (kg/d)	1.37	1.41	0.02	0.091	0.001	0.663
Milk protein yield (kg/d)	1.24^b^	1.27^a^	0.01	0.014	0.820	0.777
SCC ( × 10^4^ cells/mL)	18.04	17.81	0.11	0.186	0.131	0.976

^1^a–c Values within the same row that are marked with different lowercase letters are significantly different from each other (*P* < 0.05).

^2^Trt, treatment.

### 4.2 Effects of dietary α-amylase supplementation on blood metabolites of dairy cows

The effects of dietary α-amylase supplementation on serum metabolites in dairy cows are shown in Table 3. It can be seen from the table that the supplementation of α-amylase can significantly increase the content of amylase in the blood of dairy cows (*p* < 0.05). The effect of the experimental week on blood insulin and glucagon in dairy cows was extremely significant (*p* < 0.01), and the effect on blood amylase content showed a trend (*p* = 0.066). The interaction between the experimental treatment and the experimental week could significantly affect the blood insulin level (*p* < 0.05), but had no significant effect on other blood metabolites (*p* > 0.05).

**Table 3:** Effects of α-amylase supplementation on serum metabolites in dairy cows.

**Items**	**Treatments**		**SEM**	***p*** **-value**		
	**CON**	**AM**		**Trt** ^1^	**Time**	**Trt** × **Time**
Glucose (mmol/L)	5.24	5.88	0.34	0.216	0.160	0.278
Insulin (mIU/L)	17.06	16.42	0.36	0.237	0.001	0.021
Glucagon (pg/mL)	247.33	258.80	6.63	0.249	0.005	0.787
BUN (mmol/L)^2^	3.81	3.84	0.07	0.795	0.045	0.864
Amylase (IU/mL)	169.48^2^	181.55^1^	2.36	0.005	0.066	0.999
NEFA (μmol/L)^3^	51.79	50.69	0.51	0.159	0.170	0.959
BHBA (mmol/L)^4^	0.92	0.91	0.01	0.132	0.613	0.825

^1^Trt, treatment.

^2^BUN, blood urea nitrogen.

^3^NEFA, non-esterified fatty acids.

^4^BHBA, β-hydroxybutyric acid.

a–c Values within the same row that are marked with different lowercase letters are significantly different from each other (*P* < 0.05).

### 4.3 Effects of dietary α-amylase supplementation on nutrient digestibility of dairy cows

As shown in Table 4, dietary α-amylase supplementation could significantly increase the digestibility of starch (*p* < 0.05), and there was a trend of increased NDF digestibility (*p* = 0.063). The digestibility of OM, CP, EE, and ADF increased by 20.43%, 2.16%, 1.82%, and 6.63%, respectively (*p* > 0.05).

**Table 4:** Effects of dietary α-amylase supplementation on nutrient digestibility of dairy cows (%).

**Items^1^**	**Treatments** ^**2**^		**SEM**	***p*-value**
	**CON**	**AM**		
DM	61.14	64.72	1.40	0.217
OM	65.67	68.27	1.03	0.222
CP	72.57	74.14	2.21	0.740
EE	85.80	87.36	1.92	0.704
NDF	60.71	68.72	2.16	0.063
ADF	49.59	52.88	2.32	0.505
Starch	89.10^b^	93.38^a^	0.98	0.020

^1^DM, Dry Matter; OM, Organic Matter; CP, Crude Protein; EE, Ether Extract; ADF, Acid Detergent Fiber; NDF, Neutral Detergent Fiber.

^2^a–c Values within the same row that are marked with different lowercase letters are significantly different from each other (*P* < 0.05).

### 4.4 Effects of dietary α-amylase supplementation on rumen fermentation parameters of dairy cows

As shown in Table 5, dietary α-amylase supplementation could significantly increase the propionate concentration in the rumen (*p* < 0.05), and there was a trend of increased Total VFA (*p* = 0.085), but had no significant effect on pH, NH3–N, acetate, butyrate, and A:P (*p* > 0.05).

**Table 5:** Effects of dietary α-amylase supplementation on rumen fermentation parameters of dairy cows (%).

**Items**	**Treatments** ^**1**^		**SEM**	***p*-value**
	**CON**	**AM**		
pH	6.19	6.17	0.09	0.944
NH_3_-N	15.68	14.72	0.82	0.580
Acetic acid	58.78	59.23	1.36	0.877
Propionic acid	23.20^b^	26.13^a^	0.71	0.031
Butyric acid	12.51	13.10	0.65	0.671
TVFA	113.32	123.58	2.99	0.085
Acetic/propionic	3.15	2.96	0.15	0.562

^1^a–c Values within the same row that are marked with different lowercase letters are significantly different from each other (*P* < 0.05).

### 4.5 Effects of dietary α-amylase supplementation on the economic benefits of dairy cows

The economic benefits of dietary α-amylase supplementation for dairy cows are presented in Table 6. On the day of calculation, the milk price was 3.5 CNY/kg. The data show that compared to the control group, the supplementation of α-amylase increased the diet cost from 83.17 CNY/ (d·head) to 84.53 CNY/ (d·head), representing an increase of 1.36 CNY/ (d·head). Meanwhile, milk yield rose from 37.6 kg/d to 38.24 kg/d, an increase of 0.64 kg/d. Consequently, milk production revenue increased from 131.6 CNY/ (d·head) to 133.84 CNY/ (d·head), adding 2.24 CNY/ (d·head). The diet cost per kilogram of milk decreased from 2.2119 CNY/kg to 2.2105 CNY/kg, a reduction of 0.0014 CNY/kg. Overall, the economic benefit per cow per day increased from 48.43 CNY/ (d·head) to 49.31 CNY/ (d·head), an improvement of 0.88 CNY/ (d·head).

**Table 6:** Prices of experimental diet formulas.

**Items**	**Treatments**	
	**CON**	**AM**
Feed intake DMI (kg/d)	27.08	27.48
Milk price (CNY/kg)	3.5	3.5
Diet cost [CNY/ (cow·day)]	83.17	84.53
Milk yield (kg/d)	37.6	38.24
Milk production benefit [CNY/ (cow·day)]	131.6	133.84
Diet cost per kilogram of milk (CNY/kg)	2.2119	2.2105
Economic benefit [CNY/ (cow·day)]	48.43	49.31

## 5 Discussion

### 5.1 Effects of α-amylase on the production performance of dairy cows

Milk yield and milk quality are crucial indicators for evaluating the production performance of dairy cows. In this trial, dietary supplementation with α-amylase increased the average DMI by 0.4 kg and significantly improved milk yield compared with the CON group. This is consistent with the findings of Johnston et al. (16) on the correlation between feed intake and milk yield, which showed that each 1-h increase in feeding duration was associated with a 1.74 kg/d increase in milk yield, and each additional feeding frequency was associated with a 0.3 kg/d increase in milk yield. The research reports on the effects of α-amylase on feed intake and milk yield are not completely consistent. For example, Nozière et al. (17) found that supplementation of amylase to diets containing 20% and 30% starch did not affect body weight, feed intake, milk yield, or nitrogen metabolism in primiparous cows at 82 days of lactation, but high-starch diets increased milk protein and lactose while reducing fat and urea levels. Similarly, Weiss et al. (18) observed that in cows at 74 days of lactation, supplementation of amylase to 26% and 31% starch diets had no effects in the low-starch group, while cows on the 31% starch diet exhibited higher milk yield, fat and protein production, and feed efficiency, suggesting that high-starch diets enhance dry matter intake and energy utilization. Zilio et al. (8) found that cellulase or amylase used alone had no significant effect on milk yield or milk composition, whereas their combined use improved milk yield, lactose yield, and milk protein yield without affecting the contents of milk components. Cueva et al. (19) demonstrated that incorporating α-amylase into dairy cow silage can effectively enhance milk yield and ECM without impacting feed intake. Similarly, in the current study, the supple-mentation of α-amylase did not significantly affect feed intake but significantly in-creased milk yield, ECM, and FCM, aligning with prior research findings. The increase in milk yield may be attributed to the enhanced proportion of propionate in the rumen observed in this study. The increased proportion of propionate in late lactation may be associated with restored feed intake stimulating insulin secretion and hepatic glycogen synthesis, thereby reducing competition for circulating substrates, coupled with elevated dietary non-fiber carbohydrates providing a rapidly fermentable substrate for ruminal microbes, which activates the propionate production pathway (e.g., succinate pathway) (20). Consequently, cows have a greater availability of nutrients for milk synthesis and secretion, leading to increased milk yield. These findings indicate that α-amylase can effectively improve the production performance of dairy cows in the late lactation period and offer a novel nutritional strategy for the dairy cattle industry.

Milk fat percentage and milk protein are unique components in the milk of mammals, and their contents are closely related to milk yield. Andreozzi et al. (21) found that in a diet with 32% starch content, amylase could increase the milk yield and lactose content of dairy cows at 171 days of lactation, with a trend of increasing the total solid yield in milk but had no effect on the milk fat and protein contents and yields. However, Klingerman et al. (22) found that in a diet with 26% starch content, supplementation of α-amylase had no significant effect on the milk fat and protein contents of dairy cows at 68 days of lactation, but could significantly increase the milk fat and protein yields. In this experiment, the supplementation of α-amylase significantly increased the yield of milk protein and showed a tendency to increase milk fat percentage, total solid content, and milk fat yield, these findings are consistent with the results reported in previous studies (21, 22). Several studies have shown that milk protein yield in dairy cows increases with higher dietary starch content, particularly in both early and late lactation cows fed high-starch diets (23, 24). In late lactation, dairy cows show reduced starch-degrading bacteria abundance due to decreased energy needs and higher dietary fiber, leading to lower amylase activity while ruminal epithelial cells' adaptive starch absorption weakens, further limiting amylase efficiency (17). In this experiment, the starch content of the diet remained constant, yet the apparent digestibility of starch increased following α-amylase supplementation. This indicates that supplementing α-amylase in dairy cows during late lactation can compensate for insufficient amylase secretion, enhance starch digestibility and glucose production, thereby reducing the reliance on glucogenic amino acids for glycogen synthesis and increasing the allocation of amino acids toward protein synthesis (25–27). The increase in the total solid content and yield in milk may be related to the fact that α-amylase can break down dietary starch into small-molecule sugars (such as maltose and glucose), increasing the available energy for dairy cows, thereby promoting the synthesis of lactose, protein, and fat and in-creasing the total solid content of cow's milk (28, 29). SCC, that is, the number of somatic cells per milliliter of milk, including neutrophils, lymphocytes, macrophages, and exfoliated mammary epithelial cells, is a key indicator reflecting the health of the mammary gland and helps to identify potential mastitis. Costa et al. (30) confirmed that SCC is negatively correlated with milk yield and the percentage of lactose in milk. This study also obtained similar results. That is, supplementation of α-amylase had no significant effect on SCC, but the value of SCC in the experimental group decreased numerically, while the milk yield increased significantly, which further verified the negative correlation between SCC and milk yield. However, this study did not deeply explore the potential mechanism of SCC changes. Only numerical changes were observed, and the specific action pathway by which α-amylase affects SCC could not be determined. In the future, it is necessary to deeply study the changes of relevant immune factors and inflammatory indicators in the mammary gland tissue of dairy cows under the action of α-amylase to further reveal the molecular mechanism by which α-amylase affects SCC.

### 5.2 Effects of α-amylase on blood metabolites of dairy cows

Glucose is the main energy source in the body and reflects the glucose concentration and energy metabolism status in the body. In this study, the blood glucose level of dairy cows in the experimental group was numerically higher than that in the control group. This phenomenon indicates that α-amylase may regulate glucagon metabolism, promote glycogenolysis, the conversion of non-carbohydrate substances into glucose, and reduce the utilization of glucose by certain tissues, thereby ensuring the energy supply to key organs and tissues (29, 31). This is consistent with the result of increased milk yield in this study, further verifying the mechanism of action of α-amylase in improving dairy cow production performance. As lactation progressed, the energy negative balance in dairy cows gradually alleviated, feed intake recovered, and body reserve mobilization decreased, accompanied by a rebound in insulin levels (32). In late lactation, glucagon levels progressively declined, establishing a metabolic balance with the rising insulin (33). Reduced energy demands lead to insulin-mediated inhibition of glucagon secretion, stable feed intake maintains balanced rumen nitrogen metabolism, and decreased amino acid requirements by the mammary gland result in a decline in blood urea nitrogen (34). In this study, insulin levels gradually increased with prolonged lactation, whereas glucagon and serum urea nitrogen levels progressively decreased, findings consistent with the above mechanisms. Currently, there are relatively few studies on the effects of α-amylase on the blood metabolites of dairy cows. However, existing studies have shown that in the Arbor Acres plus chickens study (3), Aspergillus oryzae α-amylase significantly increased serum total cholesterol content and jejunal amylase activity, while Bacillus subtilis α-amylase exhibited a trend of reducing insulin levels. Some studies have also observed significant differences in the effects of different doses of amylase. For instance, Huang et al. (35) found that a low level of α-1,6-isoamylase at 300 U/kg had no significant impact on pancreatic amylase activity, whereas higher doses of α-1,6-isoamylase at 600 U/kg and 900 U/kg led to a decrease in pancreatic amylase activity. In this study, supplementation of α-amylase could significantly increase the serum amylase content, which is consistent with the previous research results and is related to the improvement of starch digestibility in this experiment. It had no significant effect on other blood metabolites, which may imply that α-amylase mainly acts on the starch decomposition process and has little impact on the overall metabolic state of dairy cows. In the energy metabolism of dairy cows, β-hydroxybutyric acid (BHB) participates in the tricarboxylic acid cycle by converting it into acetyl-CoA, thereby generating ATP (36). When dairy cows are in a negative energy balance state, changes in the concentrations of non-esterified fatty acids (NEFA) and BHB in the blood can also reflect the body's inflammatory response and oxidative stress status. It has been found that a blood BHBA concentration of ≥14.4 mg/dl (1400 μmol/L) is the most commonly used criterion for diagnosing ketosis, and when the concentration exceeds this critical point, the risk of abomasal displacement or clinical ketosis in early-lactation dairy cows is three times higher (37). Dairy cows with a blood BHBA con-centration higher than 19.4 mg/dl (about 2000 μmol/L) are at risk of reduced milk yield (38). In this study, the blood β-hydroxybutyric acid content was between 0.907-0.932 mmol/L, all within the normal range, indicating that the experimental animals had no risk of ketosis. The blood glucose and milk fat contents of dairy cows in the experimental group were higher than those in the CON group, and the contents of BHBA and NEFA in the blood were numerically lower than those in the CON group, which is also consistent with the results that the milk fat content is positively correlated with the blood and negatively correlated with BHBA and NEFA (39).

### 5.3 Effects of α-amylase on nutrient digestibility of dairy cows

Improving starch digestibility is one of the key factors in enhancing the production performance of dairy cows. Amylase can break down starch into sugars that are more easily digested and absorbed by animals, thereby improving the digestibility and utilization rate of feed. Weiss et al. (18) found that supplementation of amylase to both high-and low-starch diets can increase dry matter, organic matter, and energy metabolism efficiency, with a trend of increasing neutral detergent fiber digestibility, while its addition to high- starch diets numerically reduces protein and starch digestibility. The research results on early-lactation dairy cows show that supplementation of liquid amylase (4.4 mL/kg DM) to a diet with a starch content of about 26% can significantly increase the digestibility of organic matter, dry matter, crude protein, acid detergent fiber, and starch without affecting the feed intake, and has no effect on the digestibility of neutral detergent fiber (22). However, Nozière et al. (17) reported in early-lactation dairy cows that high-starch diets impaired NDF and ADF digestibility but boosted starch digestibility without affecting DM and OM digestibility, while amylase supplementation in such diets reduced ruminal starch digestibility and true organic matter digestibility. Hristov et al. (40) found that supplementation with 10 g/head/day of amylase and xylanase had no significant effect on the true pre-gastric digestibility of dry matter, organic matter, and neutral detergent fiber but significantly improved the apparent total tract digestibility of dry matter and organic matter, whereas their combination significantly decreased the apparent total tract digestibility of these components. Some studies have also found that α-amylase exhibits distinct effects when used in combination with other feed additives. While amylase alone has no effect on the apparent total tract digestibility in dairy cows, the combined use of amylase and proteolytic enzymes significantly improves the digestibility of starch and neutral detergent fiber, exhibiting a linear trend with the increase in proteolytic enzyme dosage (41). However, another study found that supplementation with essential oils or their mixtures with amylase tends to improve the digestibility of dry matter and crude protein, but may decrease NDF digestibility (7). This indicates that the efficacy of amylase is influenced by multiple factors. In this study, the addition of α-amylase can significantly improve the digestibility of dry matter and starch in dairy cows, and has a tendency to increase the digestibility of acid detergent fiber, organic matter, crude protein, crude fat and neutral detergent fiber. There is a certain difference from the results of previous studies, which may be related to the lactation period of dairy cows and the type of α-amylase. In this experiment, there was no negative effect on the nutrient digestibility of dairy cows under the formula starch concentration, indicating that the dietary starch level could meet the production and maintenance needs of cattle. This shows that α-amylase can promote the digestion and absorption of nutrients in feed by dairy cows, thereby improving feed utilization. In late lactation, dairy cows enter a state of positive energy balance due to declining milk yield, accompanied by enhanced insulin sensitivity. Excessive starch intake at this stage may suppress appetite via insulin, leading to a decrease in dry matter intake (42). Additionally, reduced energy requirements in late lactation and an increased fiber ratio in the diet result in decreased abundance of starch-degrading bacteria and amylase activity, thereby lowering starch digestibility (17). However, supplementation of α-amylase in the diet of late-lactation cows can effectively compensate for the deficiency of endogenous amylase, accelerating starch hydrolysis and significantly improving starch digestibility. The improvement in starch digestibility may be attributed to exogenous amylase decomposing barriers that hinder digestive enzyme access to starch granules, disrupting the crystalline structure of starch granules, promoting microbial attachment and fermentation, and increasing the contact opportunity between microbial α-amylase and starch granules, thus enhancing starch decomposition and digestibility (43). The increase in NDF digestibility may be related to the improvement of starch utilization after supplementation of amylase to the diet. When fiber-degrading bacteria such as *Butyrivibrio fibrisolvens, Fibrobacter* succinogenes, and *Prevotella ruminicola* have available starch, they will use starch as an energy source for cell wall degradation (44). In this experiment, supplementation of amylase had a trend of increasing NDF digestibility, which may be related to the promotion of the rapid growth of *Butyrivibrio fibri solvens* D1 with high fiber-degrading activity in the high-starch diet by α-amylase (45).

### 5.4 Effects of α-amylase on rumen fermentation parameters of dairy cows

Rumen fermentation parameters are important indicators for evaluating the rumen function of rumen. The rumen pH and NH3–N can reflect the stability of the rumen internal environment of ruminants and the degradation and utilization efficiency of nitrogen-containing substances by microorganisms. The ideal pH range of the rumen is 6–7, and the NH3–N content is 6.3–27.5 mg/dL (46). Excessively high or low pH and NH3–N contents are not conducive to the activity of rumen microorganisms and the utilization of nitrogen sources. In this study, the pH fluctuated between 6.17–6.19, within the normal range, and the NH3–N content was also within the normal value range, indicating that supplementation of α-amylase did not have an adverse impact on the rumen internal environment, and the rumen microbial activity of each group was normal. The production of volatile fatty acids (VFA) in the rumen of ruminants is positively correlated with energy conversion efficiency and negatively correlated with pH (47), which is consistent with the results of this study that the AM group had a higher milk yield and total volatile fatty acid (TVFA) production and a numerically lower pH than the CON group. In this study, the ammonia-nitrogen content in the AM group was numerically lower than that in the CON group, indicating that supplementation of α-amylase increased the content of easily degradable energy substances, promoted the proliferation of rumen microorganisms, accelerated the consumption of NH3–N, and promoted the synthesis of microbial protein (48). However, the content of microbial protein was not measured in this experiment, which is a shortcoming of this experiment and a key point for future research. The volatile fatty acids in the rumen mainly include acetic acid, propionic acid, and butyric acid, which account for 40%−70%, 15%−40%, and 10%−20% of the total volatile fatty acids, respectively (49). These volatile fatty acids are crucial for maintaining the stable environment of the rumen, promoting the growth of microorganisms, and improving feed digestion and absorption. Acetic acid is the main substance for synthesizing body fat and milk fat. Butyric acid is metabolized in the liver after being converted into β-hydroxybutyric acid in the rumen, providing energy for the growth and development of the body (50). During the peak lactation period in dairy cows, a high-grain diet promotes rapid starch fermentation, significantly increasing propionate proportions while reducing the acetate-to-propionate ratio, with butyrate levels remaining stable (51, 52). In contrast, during late lactation, increased dietary fiber shifts fermentation patterns toward acetate dominance (with a rising acetate-to-propionate ratio), decreases propionate proportions, and induces a slight increase in butyrate (51, 52). Correspondingly, *Ruminococcus* and *Prevotella* species, key starch-degrading bacteria, dominate the rumen microbiota during peak lactation, whereas *Fibrobacter* and *Succiniclasticum*, major fiber-degrading taxa, exhibit elevated abundance in late lactation (51, 53). Although supplementation of α-amylase in this experiment did not have a significant impact on the contents of acetic acid and butyric acid, it had a certain improving effect, which is consistent with the previous results of increased milk fat content and milk yield. The effect of amylase showed different results due to the type of amylase, the amount of amylase added and the difference of cattle. It has been reported that dairy cows supplemented with amylase have a higher ratio of acetate to propionate (54), and the propionate in the rumen mainly comes from the degradation of dietary starch by rumen microorganisms (55). Andreazzi et al. (21) and Zhao et al. (12) found that supplementation of 0.5 g/kg DM amylase and rumen-protected amylase [10, 20, and 30 g/ (d·head)] to the diet had no effect on butyric acid, acetic acid, propionic acid, and TVFA. Toseti et al. (56) obtained similar results in finishing beef cattle. However, Nozière et al. (17) found that supplementation of amylase to a 30% starch diet could significantly increase the TVFA content and the propionate molar ratio and decrease the acetate molar ratio. Felipe et al. (43) found that glucoamylase significantly improved *in vitro* dry matter digestibility, *in vitro* starch digestibility, and gas production, linearly decreased total volatile fatty acids, and increased isovalerate, whereas Thermolysin (metal endopeptidase) and Neutral protease (metal endopeptidase) had no effect on total volatile fatty acids, acetate, and propionate. After supplementation of α-amylase in this experiment, the rumen propionate increased significantly, and there was a trend of increasing TVFA. The acetate-to-propionate ratio was numerically lower than that in the CON group. This may be related to the fact that the experimental animals in this study were dairy cows in the late lactation period. In the rumen of dairy cows during late lactation, the dominant phyla are Bacteroidetes, Proteobacteria, and Firmicutes, with the dominant families being *Succinivibrionaceae* and *Prevotellaceae*, and these microorganisms break down starch into propionate and other volatile fatty acids via their rich enzyme systems and metabolic pathways (57–59). Propionate serves as a precursor for glucose synthesis in ruminants, generating glucose via the gluconeogenesis pathway to supply energy, and the acetate/propionate ratio is closely related to energy utilization efficiency, with a lower ratio indicating higher efficiency (20). In this study, the AM group of dairy cows had higher blood glucose content and milk yield, which is consistent with the previous research results. The increase in plasma glucose concentration caused by amylase may be the result of increased ruminal fermentation of starch and net absorption of propionate for gluconeogenesis in the liver (21). The improvement of TVFA in the AM group may be related to the improvement of dietary quality by supplementation of α-amylase and the increase in feed intake (60). The acetate/propionate ratio represents the rumen fermentation type. In this experiment, the decreased acetate/propionate ratio and increased molar proportion of propionate indicated a shift toward propionate-type fermentation in the rumen, which provides more energy for dairy cows. The rumen acetate/propionate ratio of the AM group was lower than that of the CON group. Therefore, the AM group had higher energy utilization efficiency. This implies that α-amylase may promote the utilization of starch and energy production in dairy cows by regulating the rumen fermentation pattern.

## 6 Conclusions

Supplementing the diet of Holstein dairy cows in late lactation with 15 g/d of α-amylase enhanced ruminal fermentation, as evidenced by increased concentrations of propionate and total volatile fatty acids. These improvements were accompanied by elevated digestibility of dry matter, starch, and acid detergent fiber. Additionally, this dietary intervention led to increased blood α-amylase activity and improved blood metabolic profiles. Collectively, these changes contributed to increased milk yield and improved milk composition. Future research should focus on elucidating the specific mechanisms underlying these effects and exploring the long-term impacts of α-amylase supplementation on dairy cow health and productivity.

## Data Availability

The original contributions presented in the study are included in the article/Supplementary material, further inquiries can be directed to the corresponding author/s.
